# Effect of Shenqi compound on inflammatory markers and glycemic measures among diabetes mellitus: A protocol for systematic review and meta-analysis

**DOI:** 10.1097/MD.0000000000020736

**Published:** 2020-07-02

**Authors:** Yan Yang, Xiaoxu Fu, Wen Zhong, Zhipeng Hu, Yuan Tian, Hui Zhou, Hong Gao, Chunguang Xie

**Affiliations:** a Chengdu University of Traditional Chinese Medicine; b Affiliated Hospital of Chengdu University of Traditional Chinese Medicine, Sichuan, China.

**Keywords:** diabetes, glycemic measures, inflammatory markers, protocol, Shen-qi compound, systematic review and meta-analysis

## Abstract

**Background:**

Diabetes is a chronic disease characterized by chronic hyperglycemia, absolute or relative deficiency of insulin secretion, and chronic inflammation. Shenqi compound (SC) is a traditional Chinese medicine formula widely used in the treatment of diabetes and diabetic complications. Although many randomized clinical trials have proved that SC can benefit a lot from diabetes and its complications, the systematic evaluation of the effect of SC on diabetic blood glucose control and inflammatory markers has not yet appeared. The purpose of this study is to provide evidence that the therapeutic effect of SC on diabetes and its multiple system complications is related to its control of blood glucose and inflammatory mediators.

**Methods:**

Three English database and 4 Chinese medical databases will be searched from its inception to May 2020. Then 2 methodological trained researchers will screen the qualified articles by reading the title, abstract, and full texts according to an established inclusion and exclusion criteria. The assessment of risk of bias will be conducted by using the Cochrane collaboration's tool. We will conduct meta-analyses for fasting blood glucose, postprandial blood glucose, glycated hemoglobin, tumor necrosis factor, C-reactive protein or high-sensitivity C-reactive protein, and other outcomes. The heterogeneity of data will be evaluated by Cochrane *X*^2^ and *I*^2^ tests. Subgroup analysis will also be carried out. We will conduct sensitivity analysis to evaluate the stability of the results, funnel plot analysis, and Egger test to evaluate the publication bias, and assessment for the quality of evidence by the grading of recommendations assessment, development, and evaluate system.

**Results:**

The results of our research will be published in a peer-reviewed journal.

**Conclusion:**

In this study, we will systematically evaluate the influences of SC on glycemic measures and inflammatory markers of diabetes mellitus. Our research is supposed to provide evidence-based support for clinical practice.

**Registration number:**

INPLASY202040179.

## 1 Introduction

Diabetes mellitus is a syndrome made up of several diseases with similar symptoms, signs, and complications with different etiologies.^[1]^ According to the different etiology and pathology, it can be divided into type 1 diabetes, type 2 diabetes, and special type diabetes. The basic feature of diabetes is chronic hyperglycemia caused by absolute or relative insufficiencies of insulin.^[2]^ Blood glucose fluctuation is considered to be an important predictor of chronic vascular complications associated with diabetes.^[3]^ Hyperglycemia and insulin resistance usually leads to metabolic disorders of nutrients such as glucose, fat, and protein in diabetic patients. Insulin resistance particularly is an important cause of obesity in diabetic patients. Obesity is closely related to the inflammatory state of the body by increasing the expression of inflammatory factors.^[4]^ Interleukin 6 (IL-6), tumor necrosis factor-α are shown to be positively correlated with increased adiposity^[5]^ and C-reactive protein (CRP) increases with visceral obesity.^[6]^ Shenqi compound is a traditional Chinese medicine compound established under the guidance of traditional Chinese medicine theory. In China, the application of traditional Chinese herbal medicine in DM has a long history and rich experience.^[7]^ Shenqi compound has been used in clinical settings for 16 years.^[8]^ It contains 8 herbal medicine: Radix et Rhizoma Ginseng (rénsh”en), Radix AstragaliPraeparata cum Melle (huáng qí), Radix et RhizomaSalviaeMiltiorrhizae (dānsh”en), Radix et RhizomaRhei (dà huáng), RhizomaDioscoreae (huáishānyào), Radix Trichosanthis (tiānhuā fěn), Radix Rehmanniae (dì huáng), FructusCorni (shānzhuyu).^[7]^ Many animal studies have proved that Shenqi compound can improve insulin resistance, improve blood glucose, improve inflammatory markers, improve lipid metabolism so as to treat diabetes and its complications.^[9–12]^ In addition, clinical trials also found that it can improve blood glucose, insulin sensitivity, and chronic inflammatory state.^[13–16]^ Although it has been found that Shenqi compound has many benefits in improving blood glucose, fat metabolism, and inflammatory markers in diabetes. The systemic evaluation of Shenqi compound on the regulation of blood glucose and inflammatory markers in diabetes mellitus and its complications has not yet appeared. The purpose of this study is to systematically evaluate the accurate effect of Shenqi compound on the regulation of diabetic blood glucose and inflammatory markers.

## 2 Methods and analysis

### 2.1 Study registration

A prospective protocol regarding the detailed search strategy and methods of data analysis was prepared according to the preferred reporting items for systematic reviews and meta-analysis of observation studies in epidemiology recommendations for study reporting. This systematic review and meta-analysis protocol is reported according to the preferred reporting items for systematic reviews and meta-analysis protocols checklist.^[17]^

### 2.2 Inclusion and exclusion criteria

Population: Adults patients with an established DM diagnose will be included in our research. There is no limitation about region, sex, and age of patients.

Intervention: Studies that use Shengqi compound as a major intervention in experimental group will be included. The control group can use any other medicines or placebo. If the authors use combination therapy of Shenqi compound and other medicines in experimental group, these studies will be excluded.

Outcomes: Only those studies that report detailed data about DM-related outcomes will be included in our research. The definition of diabetes can be based on any accepted diagnostic criteria [such as American Diabetes Association (ADA) guidelines]. Studies whose outcomes are not directly related to DM will be excluded. Those studies including abstract articles, conference articles, review articles, systematic review, and meta-analysis will be excluded. For conference articles and abstract articles, we will further look for whether there are any original articles that have made further reports on their research.

Study design: We will only include prospective randomized controlled trials (RCTs). Observational studies will be excluded. Similarly, retrospective studies will not be included in our research.

### 2.3 Outcomes

Main outcome: glycated hemoglobin; tumor necrosis factor-α.

Secondary outcome: postprandial blood glucose; fasting blood glucose; CRP or high-sensitivity-CRP; IL-6; IL-4.

### 2.4 Study search

We will search 3 English database including PubMed, Embase, the Cochrane Library Central Register of Controlled Trials, and 4 Chinese databases including China National Knowledge Infrastructure database, Wanfang Data Knowledge Service Platform, the VIP information resource integration service platform, China Biology Medicine Disc from their inception to May 2020 with a language limitation of English and Chinese. In addition, the Chinese Clinical Trial Registry and ClinicalTrials.gov will be searched to ensure that no clinical studies are missed. We will also conduct a manual search in the library of Chengdu University of Chinese Medicine in case of any missing literature.

We will retrieve all the literature related to Shenqi compound and then screen it according to inclusion and exclusion criteria. The search terms used will be as follows: “shenqifufang,” “shenqi compound,” “SHEN-QI compound,” “shenqi formula,” “shenqi compound formula,” “Shen-Qi Compound formula,” “Shen-Qi formula.” Two authors will search and screen all the citations independently. An example of search process is presented in Table 1.

**Table 1:**
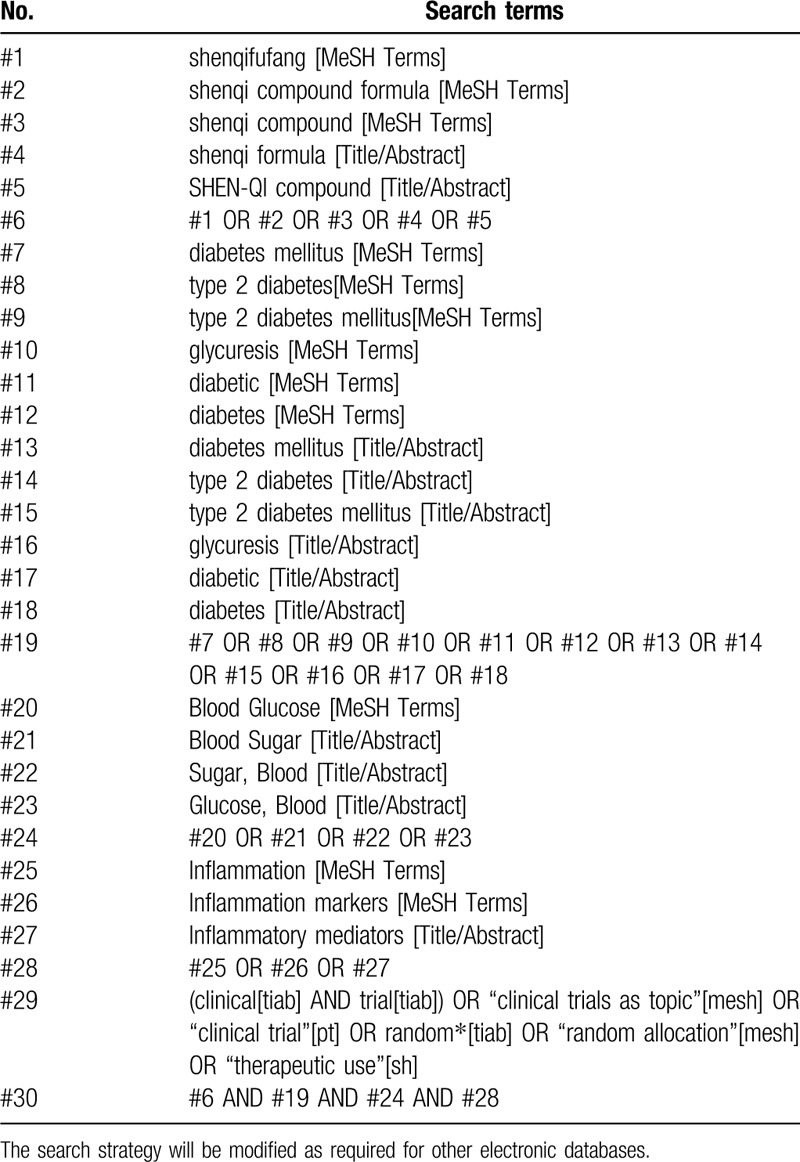
Search strategy for PubMed.

### 2.5 Study selection

**Figure 1:**
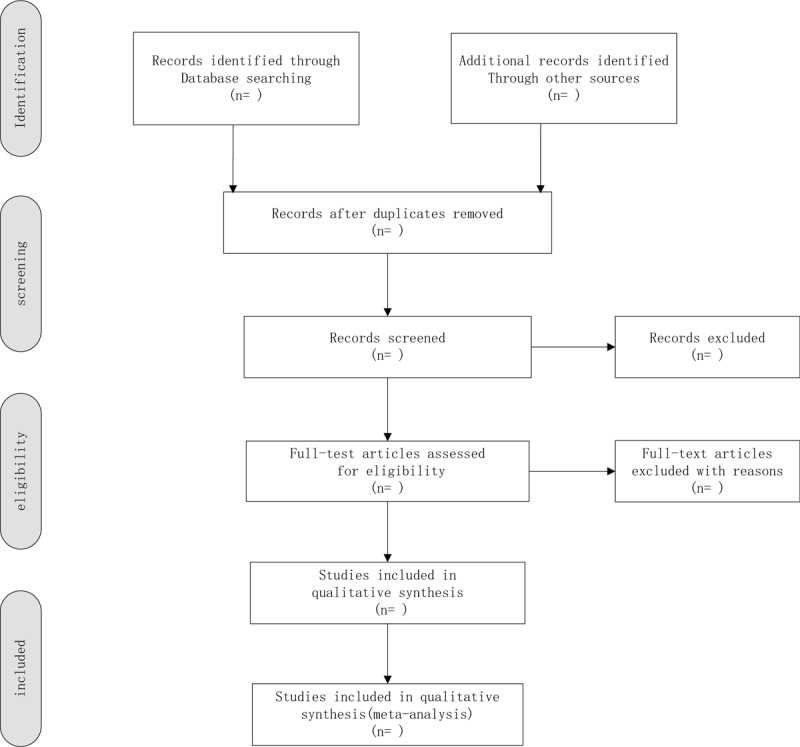
Flow chart of the study selection.

According to the inclusion criteria, 2 independent reviewers will evaluate all titles and abstracts to exclude papers that are not considered relevant. The remaining provisions will be included in a further assessment. Reviewers will carefully examine the full text of each potentially relevant article. The process of study identification and exclusion will be described with preferred reporting items for systematic reviews and meta-analysis flow chart. Differences in research options will be resolved through consultation (Fig. 1).

### 2.6 Data extraction

The data of those qualified articles will be extracted into Microsoft Excel according to a predefined form for further analysis. For those studies with incomplete data, we will contact the authors for more detailed information. If we cannot contact the authors or the authors refuse to provide detailed information, then the document will be excluded. The extracted data is as follows: title, the publication country, the first authors of the article, time of publication, baseline information of participants, interventions in experimental group, interventions in control group, time and dose of treatment, course of disease, number of patients in each group, ages and sex of patients and outcomes.

### 2.7 Risk of bias assessment

The risk of bias of included studies will be assessed by using the Cochrane collaboration's tool. In this tool, the risk of bias of a study is assessed from 7 aspects: random sequence generation (selection bias), allocation concealment (selection bias), blinding of participants and personnel (performance bias), blinding of outcome assessment (detection bias), incomplete outcome data (attrition bias), selective reporting (reporting bias), other bias.^[18]^ Each item is classified as “Low risk,” “High risk,” or “Unclear risk.” If the experimental process is not described in detail in the article, we will contact the authors by E-mail for more methodological information. Two reviewers will conduct the risk of bias assessment independently and any disagreements will be solved by consensus.

### 2.8 Data analysis

Data analysis will be conducted in Review Manager Version 5.3 and Stata 14.0 software. For the binary variable, the effect size will be represented as risk ratio and 95% confidence interval and a Der-Simonian-Laird method will be used to calculate them. Continuous data will be reported as an average difference from their 95% confidence intervals. *I*^2^ statistics will be used to test the statistical heterogeneity of the test. 75%, 50%, or 25% of *I*^2^ statistics indicate high, medium, and low heterogeneity, respectively. The statistical heterogeneity is considered substantial when *P* < .05 and *I*^2^ > 50% and the random-effect model will be applied to pool data. If there is no significant heterogeneity (*P* > .05 and *I*^2^ < 50%), then the fixed- effect model will be used to calculate the effect size. If quantitative synthesis is not appropriate due to substantial heterogeneity, then systematic review will be conducted and the results will be presented with tables and figures.

### 2.9 Investigation of heterogeneity

If there is substantial heterogeneity between studies, then we will conduct subgroup analysis to explore the heterogeneity. Sub groups were discussed according to age (18–45 years and at least 45 years), gender, and body mass index (25–29.9 kg/m^2^ and ≥30 kg/m^2^). In addition, we can also conduct subgroup analysis according to different complications of diabetes.

### 2.10 Sensitivity analysis

To ensure the stability of the results, we will conduct sensitivity analysis of the results by excluding each of the studies included in the analysis one by one. If there is one or more very large study, we will repeat the analysis excluding them to determine how much they dominate the results.

### 2.11 Reporting bias assessment

The integrity of the included studies is mainly measured by reporting bias, of which publication bias is the most common. Therefore, this study will identify report bias by publication bias assessment. A funnel plot will be drawn to investigate the publication bias. Funnel plot will be asymmetric when publication bias exists, such as when research with small sample and no statistically significant results are not published. The more obvious the asymmetry of funnel plot is, the more likely there is publication bias.^[19]^ And then Egger test will be conducted for statistical assessment the publication bias. The publication bias is considered to exist if *P* < .05.^[20]^

### 2.12 Quality of evidence

Finally, this study will evaluate the quality of evidence for each outcome through grading of recommendations assessment, development, and evaluate system. RCTs will be defined as high-level evidence and observational studies as low-level evidence in grading of recommendations assessment, development, and evaluate system system. Researchers can down-grade the quality of evidence in RCTs from a high level to a moderate or lower level depending on whether there are factors affecting the quality of evidence.

### 2.13 Patient and public involvement

There is no patient and public involved in this study.

### 2.14 Ethics and dissemination

Ethical approval is not needed for this meta-analysis. This study comprehensively evaluates the existing research evidence of SC and can provide evidence-based medical support for clinical workers. The results of our research will be published in a peer-reviewed journal.

## 3 Discussion

There are many complications in diabetes. In fact, they all share the same pathogenic factors and pathological mechanism. Chronic hyperglycemia will damage the function of mitochondria, and the mitochondrial dysfunction will further aggravate the metabolic disorder, which will lead to the accumulation of adipose tissue and the production of inflammatory mediators. Hyperglycemia is an important factor affecting mortality. In 2017, the total number of deaths at all ages due to high fasting plasma was 6.5 million, of which type 2 diabetes accounted for 1 million deaths.^[21]^ In this study, the blood glucose and inflammatory markers of Shenqi compound in regulating diabetes and its complications were systematically evaluated. And it is very important to improve the metabolic disorder and inflammation caused by hyperglycemia and insulin resistance. So our research will provide specific and high quality evidence for Shenqi compound in the treatment of diabetes and its complications. The limitation of this study is to exclude some animal studies, which may have an impact on the sample size included in the study. Moreover shenqi compound is a traditional Chinese medicine compound. Although its curative effect is very outstanding, the research is mainly concentrated in China, maybe it will lead to heterogeneity influenced by ethic. Therefore, the randomized clinical trials for different ethnic groups should be carried out later.

### 3.1 Amendments

If any modification is required, we will update our protocol to include any changes in the entire research process.

## Author contributions

**Conceptualization:** Yan Yang, Xiaoxu Fu, Chunguang Xie.

**Data curation:** Wen Zhong.

**Formal analysis:** Yan Yang, Xiaoxu Fu, Chunguang Xie.

**Funding acquisition:** Hong Gao, Chunguang Xie.

**Investigation:** Wen Zhong, Yuan Tian.

**Methodology:** Xiaoxu Fu, Zhipeng Hu, Hui Zhou, Hong Gao, Chunguang Xie, Yan Yang

**Project administration:** Chunguang Xie.

**Resources:** Yan Yang, Chunguang Xie.

**Software:** Zhipeng Hu, Yuan Tian, Hong Gao, Yan Yang, Xiaoxu Fu.

**Supervision:** Xiaoxu Fu.

**Writing – original draft:** Yan Yang.

**Writing – review & editing:** Hui Zhou, Chunguang Xie.

## References

[1] Guthrie RA Guthrie DW Pathophysiology of diabetes mellitus. Crit Care Nurs Q 2004;27:113–25. 15137354 10.1097/00002727-200404000-00003

[2] DeFronzo RA Ferrannini E Zimmet P International Textbook of Diabetes Mellitus, 2 Volume Set, 4th Edition. 2015:1–17.

[3] Wang JZ Liu SQ Jiang X Effect and mechanism of blood glucose fluctuations on macrovascular disease in patients with diabetes. Med Rev 2019;25:4493–7.

[4] Levine TB Levine AB Metabolic Syndrome and Cardiovascular Disease, Second Edition. 2006:192–227.

[5] Dandona P Ghanim H Mohanty P The metabolic syndrome: linking oxidative stress and inflammation to obesity, type 2 diabetes, and the syndrome. Drug Dev Res 2006;67:619–26.

[6] Després J-P Lemieux I Abdominal obesity and metabolic syndrome. Nature 2006;444:881–7. 17167477 10.1038/nature05488

[7] Hu Z Yang M Xie C Efficacy and safety of Shenqi compound for the treatment of diabetic macroangiopathy. Medicine 2020;99:e19682. 32282722 10.1097/MD.0000000000019682 PMC7220121

[8] Zhu Q Kang J Xu G Traditional Chinese medicine Shenqi compound to improve lower extremity atherosclerosis of patients with type 2 diabetes by affecting blood glucose fluctuation. Medicine 2020;99:e19501. 32176092 10.1097/MD.0000000000019501 PMC7440330

[9] Zhang Y Wang H Fu X Effects of YangyinYiqiHuoxue method on oxidative stress and nitrification stress injury in diabetic GK rats. Chin J Tradit Chin Med 2017;35:2066–9.

[10] Zhang HM Chen SW Xie CG Effects and mechanism of ShenQi compound recipe on inflammation maker in GK rats. J Chin Med Mat 2006;29 (3.):249–53. 16850723

[11] Liu Y Kang J Gao H Exploration of the effect and mechanism of ShenQi compound in a spontaneous diabetic rat model. EndocrMetab Immune Disord Drug Targets 2019;19:622–31. 10.2174/1871530319666190225113859 30799801

[12] Gao H Duan Y Fu X Comparison of efficacy of SHENQI compound and rosiglitazone in the treatment of diabetic vasculopathy analyzing multi-factor mediated disease-causing modules. PLoS One 2018;13:e0207683. 30521536 10.1371/journal.pone.0207683 PMC6283585

[13] Fu X Xie C Clinical observation of 57 cases of type 2 diabetic macrovascular disease treated with Shenqi compound prescription. J Tradit Chin Med 2013;54:1297–300.

[14] Zhang X Liu Y Xiong D Insulin combined with Chinese medicine improves glycemic outcome through multiple pathways in patients with type 2 diabetes mellitus. J Diabetes Investig 2015;6:708–15. 10.1111/jdi.12352 PMC4627549 26543546

[15] Han X Clinical observation on 100 cases of type 2 diabetic macrovascular disease treated by Shenqi compound prescription. Abstracts World's Latest Med Inf 2017;17:196–7.

[16] Zhao X Shenqi compound prescription combined with viglistine in the treatment of diabetic macrovascular disease. J Mod Integr Chin Western Med 2018;27:2804–7.

[17] Moher D Shamseer L Clarke M Preferred reporting items for systematic review and meta-analysis protocols (PRISMA-P) 2015 statement. Syst Rev 2015;4:1. 25554246 10.1186/2046-4053-4-1 PMC4320440

[18] Higgins JPT Thomas J Chandler J Cochrane Handbook for Systematic Reviews of Interventions version 6.0 (updated July 2019). Cochrane, 2019. Available from www.training.cochrane.org/handbook.

[19] Peters JL Sutton AJ Jones DR Contour-enhanced meta-analysis funnel plots help distinguish publication bias from other causes of asymmetry. J Clin Epidemiol 2008;61:991–6. 18538991 10.1016/j.jclinepi.2007.11.010

[20] Egger M Davey Smith G Schneider M Bias in meta-analysis detected by a simple, graphical test. BMJ 1997;315:629–34. 9310563 10.1136/bmj.315.7109.629 PMC2127453

[21] GBD 2017 Causes of Death Collaborators. Global, regional, and national age-sex-specific mortality for 282 causes of death in 195 countries and territories, 1980–2017: a systematic analysis for the global burden of disease study 2017. Lancet. 2018;392:1736–88. 10.1016/S0140-6736(18)32203-7 PMC6227606 30496103

